# Atrial structure, function and arrhythmogenesis in aged and frail mice

**DOI:** 10.1038/srep44336

**Published:** 2017-03-14

**Authors:** Hailey J. Jansen, Motahareh Moghtadaei, Martin Mackasey, Sara A. Rafferty, Oleg Bogachev, John L. Sapp, Susan E. Howlett, Robert A. Rose

**Affiliations:** 1 Department of Physiology and Biophysics, Faculty of Medicine, Dalhousie University, Halifax, Nova Scotia, Canada; 2 Division of Cardiology, Faculty of Medicine, Dalhousie University, Halifax, Nova Scotia, Canada; 3 Department of Pharmacology Faculty of Medicine, Dalhousie University, Halifax, Nova Scotia, Canada; 4 School of Biomedical Engineering, Faculty of Medicine, Dalhousie University, Halifax, Nova Scotia, Canada

## Abstract

Atrial fibrillation (AF) is prevalent in aging populations; however not all individuals age at the same rate. Instead, individuals of the same chronological age can vary in health status from fit to frail. Our objective was to determine the impacts of age and frailty on atrial function and arrhythmogenesis in mice using a frailty index (FI). Aged mice were more frail and demonstrated longer lasting AF compared to young mice. Consistent with this, aged mice showed longer P wave duration and PR intervals; however, both parameters showed substantial variability suggesting differences in health status among mice of similar chronological age. In agreement with this, P wave duration and PR interval were highly correlated with FI score. High resolution optical mapping of the atria demonstrated reduced conduction velocity and action potential duration in aged hearts that were also graded by FI score. Furthermore, aged mice had increased interstitial fibrosis along with changes in regulators of extracellular matrix remodelling, which also correlated with frailty. These experiments demonstrate that aging results in changes in atrial structure and function that create a substrate for atrial arrhythmias. Importantly, these changes were heterogeneous due to differences in health status, which could be identified using an FI.

Atrial fibrillation (AF) is the most common sustained arrhythmia^1^. It is an enormous clinical problem that is projected to affect up to 30 million people in North American and Europe by the year 2050^2^. AF is associated with substantial morbidity and mortality, is prevalent in heart failure (which is worsened when coexisting with AF)^3^ and is a major risk factor for ischemic stroke^4^,^5^. It is well known that the prevalence of AF increases with age^4^,^6^. Furthermore, epidemiological studies demonstrate that the progression of AF from persistent or paroxysmal forms to permanent AF is also associated with increasing age^7^.

Although it is well appreciated that the frequency of AF development increases with age it is critical to recognize that not all individuals age at the same rate and an individual’s health status can vary from fit to frail regardless of chronological age^8^,^9^,^10^. This is because people accumulate health deficits, which manifest as frailty, at different rates and for different reasons^11^. People that age rapidly are described as frail, while those that age more successfully are fitter. As individuals become clinically frail the risk of adverse health outcomes increases^12^,^13^. Frailty is very common in patients with cardiovascular disease^14^.

A well-established clinical method to quantify frailty is to measure deficit accumulation using a frailty index (FI)^8^,^11^. In this approach, deficits in health status may include signs, symptoms, diseases, disabilities and/or laboratory abnormalities. The FI score for an individual is represented by the proportion of deficits present in that individual, whereby the number of health deficits present in each individual is counted and divided by the total number of items measured. Accordingly, FI scores can range from 0 (no deficits present; least frail) to 1 (all deficits present; most frail). We have developed a non-invasive 31 item FI based on deficit accumulation for use in animal models (i.e. mice)^15^,^16^,^17^. Using this FI we have demonstrated that the relationships between FI score and age are very similar in mice and humans. Furthermore, we have recently used our FI approach to study the impacts of age and frailty on ventricular myocyte^18^ and sinoatrial node^19^,^20^ function in mice. Collectively, our prior work demonstrates that our FI approach is a powerful new tool to study aging-related changes in the heart.

While the clinical links between age and AF are well established, very few studies have investigated the cellular and molecular mechanisms that create a substrate for AF in aged individuals and no studies have evaluated how atrial function is related to frailty. Our development of a method for quantifying frailty in mice enables detailed studies, including at the cellular and molecular levels, of age-related cellular dysfunction. Indeed, it has been proposed that alterations at the cellular and subcellular levels may scale up and give rise to deficits that can be detected clinically^21^. This represents an essential area of investigation that will provide much needed insight into the biology of frailty, which is not well understood. Accordingly, the purpose of this study was to determine how atrial structure and function are affected by age and frailty in mice using our deficit accumulation approach. Our experiments demonstrate that atrial arrhythmogenesis and changes in atrial function are present in aged mice and that these occur in association with electrical and structural remodelling in the atria. We also show that alterations in atrial structure and function, including at the molecular level, are strongly predicted by individual health status as assessed using a frailty index.

## Methods

An expanded Methods section is available in the Supplementary data.

### Mice and frailty assessment

This study utilized young (13.1 ± 3.0 weeks; *n* = 80) and aged (101.8 ± 10.1 weeks; *n* = 110) male C57BL/6 mice, which were purchased at the age of 3–4 weeks and housed in our animal care facility until used experimentally. All experimental procedures were approved by the Dalhousie University Committee for Laboratory Animals and followed the guidelines of the Canadian Council on Animal Care.

Frailty was assessed in all mice immediately prior to experimental use using our non-invasive 31 item frailty index, which is based on established clinical signs of health deterioration in mice^15^,^22^ as we have done previously^19^. Specific clinical assessments of the integument, musculoskeletal, vestibulocochlear/auditory, ocular, nasal, digestive, urogenital and respiratory systems were made. We also assessed mice for signs of discomfort and measured both body surface temperature and body mass. Each of the 31 items that were assessed is listed in our published frailty assessment form (Supplemental Table 1)^15^. To score deficit accumulation, each trait was given a score of 0 (no sign of deficit), 0.5 (mild deficit) or 1 (severe deficit). Deficits in body mass and body temperature were scored based on deviation from the mean of young or aged mice^15^,^19^. For each animal the scores for each trait were summed and the total was divided by the number of items measured (i.e. 31) to provide a frailty index score between 0 (least frail) and 1 (most frail). Individuals performing functional measurements did not know the FI score of the mice until after data were analyzed.

### *In vivo* electrophysiology and programmed stimulation

Electrophysiology and arrhythmia studies were performed in anesthetized mice (2% isoflurane) using 30 gauge subdermal needle electrodes (Grass Technologies) to record body surface (lead II) ECGs. In addition, a 1.2 French octapolar electrophysiology catheter was inserted in the heart via the right jugular vein, as we have described previously^23^,^24^. P wave duration and PR interval were measured from surface ECGs. Atrial (AERP) and atrioventricular node (AVERP) effective refractory periods were measured using S1/S2 stimulation protocols in which the heart was paced at a cycle length of 100 ms (S1) followed by an extra stimulus (S2) at progressively shorter cycle lengths. Inducibility of AF was measured using burst pacing in the right atrium. AF was defined as a rapid and irregular atrial rhythm lasting a minimum of 1s on the surface ECG. AF was categorized into three groups: <5s (brief); 5–30s (non-sustained); >30s (sustained). Further details are provided in the Data Supplement.

### High resolution optical mapping

To investigate patterns of electrical conduction in the atria we used high resolution optical mapping in atrial preparations as we have described previously^23^,^24^,^25^. Optical mapping was performed using the voltage sensitive dye di-4-ANEPPS (10 μM). Contractile activity was suppressed using blebbistatin (10 μM)^26^. All analyses were performed using custom software. Details are provided in the Data Supplement.

### Collagen staining and collagen assay

Interstitial collagen was assessed using picrosirius red (collagen) and fast green (myocardium) staining of paraffin embedded sections (3 μm) through the right and left atria. The level of fibrosis was quantified using ImageJ software as previously described^23^,^24^. Total collagen content was measured using a hydroxyproline assay (Sigma-Aldrich) according to kit instructions. These assays were performed using the right and left atrial appendages.

### Quantitative PCR

Quantitative gene expression was measured in the right and left atria as we have done previously^23^,^27^,^28^. Intron spanning primers (Supplemental Table 2) were designed for collagen I (*col1a*), collagen III (*col3a*), matrix metalloproteinase 2 (MMP2), MMP9, tissue inhibitor of metalloproteinase 1 (TIMP1), TIMP2, TIMP3, TIMP4, transforming growth factor β (TGFβ) and connective tissue growth factor (CTGF). β-actin (*Actb*) and GAPDH were used as reference genes. Experimental protocols are described in the Data Supplement.

### Statistical analysis

All data are presented as box and whisker plots (including median, upper and lower quartiles, upper and lower maximums) or means ± SD. Data were analyzed using Student’s *t*-test, two-way ANOVA with Tukey’s posthoc test or Fisher’s exact test, as indicated in each figure legend, to detect differences in young vs. aged mice. To assess differences as a function of frailty we performed linear regression analysis and used Pearson’s correlation to obtain correlation coefficients. *P* < 0.05 was considered significant.

## Results

### Assessment and quantification of frailty in mice

**Figure 1:**
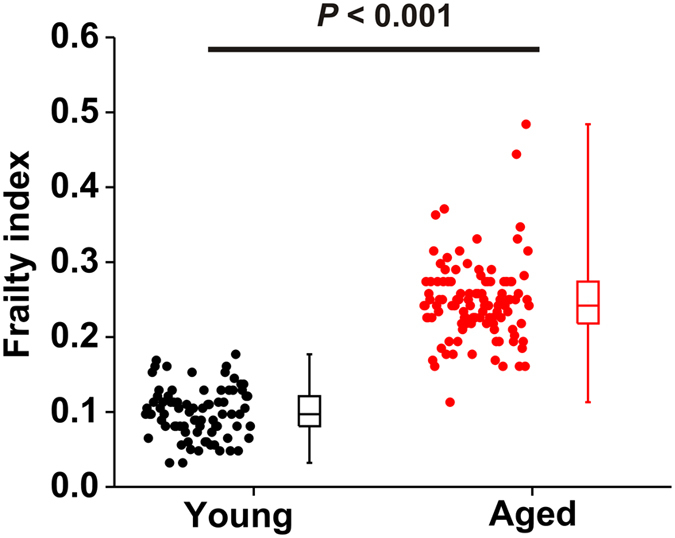
Frailty index scores in young and aged mice. Summary data illustrating FI scores in all young (n = 80) and aged (n = 110) mice used throughout this study. Data analyzed by Student’s *t*-test.

All mice utilized in this study underwent frailty assessment using our non-invasive FI^15^,^16^ resulting in the generation of an FI score for each individual. Young mice had an average FI score of 0.11 ± 0.01 while aged mice had an average FI score of 0.25 ± 0.01 (*P* < 0.001; Fig. 1). It is apparent from the data in Fig. 1 that mice of similar chronological age can have a range of FI scores. This is especially true in the aged mice and is indicative of differences in health status regardless of chronological age. Furthermore, it is important to note that while aged mice had a higher average FI score, there is overlap in these FI scores between young and aged mice even though they differ substantially in chronological age. Thus, by assessing frailty, we are able to identify differences in health status among different age groups as well as within a given age group, which is consistent with our previous work in this area^19^.

### Occurrence of atrial fibrillation and atrioventricular node block *in vivo*

**Figure 2:**
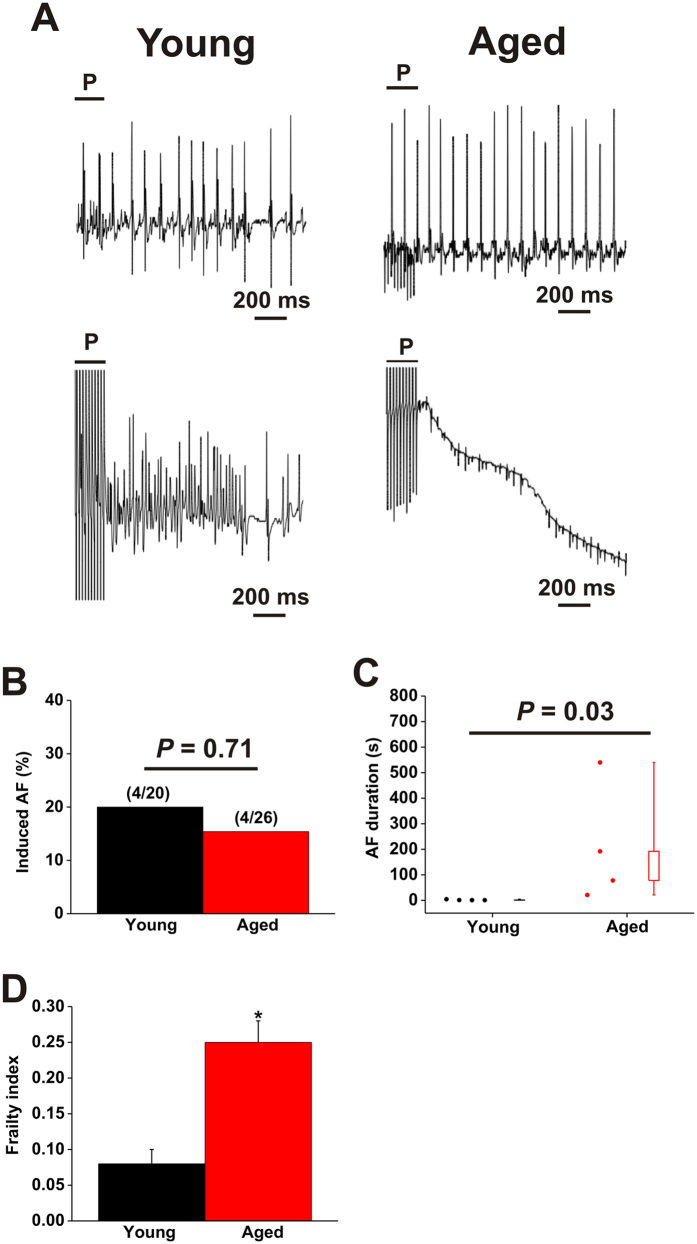
Susceptibility to induced atrial fibrillation in young and aged mice. (**A**) Representative body surface (top) and intracardiac atrial (bottom) ECGs illustrating the induction of AF following burst pacing (P) in young and aged mice. Note that the young mouse spontaneously returned to sinus rhythm after 1.1s of AF while the aged mouse remained in AF over the same time period. In this example, the aged mouse was in AF for a total of 21.3s. (**B**) Summary of inducibility of AF in young and aged mice. Numbers in parentheses indicate the number of mice that were induced into AF following burst pacing. Data analyzed by Fischer’s exact test. (**C**) Summary data illustrating the duration of AF in young (*n* = 4) and aged (*n* = 4) mice that were induced into AF. Data analyzed by Student’s t-test. (**D**) Frailty index scores in young and aged mice that were induced into AF. *P < 0.05 vs. young by Student’s t-test.

As AF is a common and serious problem in the aged population we began by assessing the occurrence and duration of AF during burst pacing in anesthetized mice (Fig. 2A). Although we did not observe a difference in overall susceptibility to AF in young vs. aged mice (Fig. 2B) we found that the duration of AF, when induced, was longer (*P* = 0.03) in aged mice compared to young mice (Fig. 2C). In fact, all AF in young mice was non-sustained, lasting less than 5s before spontaneously reverting back to sinus rhythm (Fig. 2C; Supplemental Table 3). In contrast, when AF was induced in aged mice it was long lasting. Specifically, one mouse had AF that lasted 21.3s, while the remainder all demonstrated sustained AF lasting longer than 30s and up to several minutes (Fig. 2C, Supplemental Table 3). The average FI score in young mice demonstrating AF was 0.08 ± 0.02 while the aged mice demonstrating AF had a higher (*P* < 0.05) average FI score of 0.25 ± 0.03 (Fig. 2D) indicating that mice with higher FI scores were more likely to experience sustained AF.

**Figure 3:**
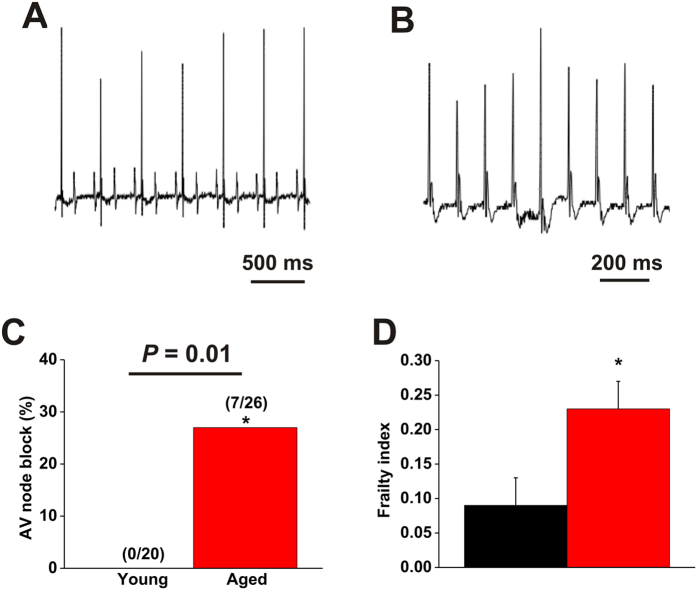
Occurrence of atrioventricular node block in aged mice. (**A**) Representative surface ECG illustrating an example of 2:1 AV node block in an aged mouse. (**B**) Representative surface ECG illustrating an example of complete AV node block in an aged mouse. (**C**) Summary data illustrating the frequency of occurrence of AV node block in young vs. aged mice. Numbers in parentheses indicate the number of mice that demonstrated AV node block. Data analyzed by Fischer’s exact test. (**D**) Frailty index scores in young and aged mice used in assessment of AV node block. *P < 0.05 vs. young by Student’s t-test.

Further evidence of a substrate for AF and abnormal conduction in aged mice was obtained by measuring the occurrence of AV node block (Fig. 3A). No AV node block was observed in young mice (0/20 mice). In contrast, 27% of aged mice (7/26 mice) displayed spontaneous AV node block (either 2:1 or complete AV node block; Fig. 3C). Average FI score in the aged mice used to assess incidence of AV node block was 0.23 ± 0.04, which was higher (*P* < 0.05) than the average FI score of 0.09 ± 0.04 (Fig. 3D) in the young mice used in this experiment, further suggesting that mice with higher FI scores are more susceptible to arrhythmias.

### Effects of age and frailty on conduction in the atria and through the atrioventricular node *in vivo*

**Figure 4:**
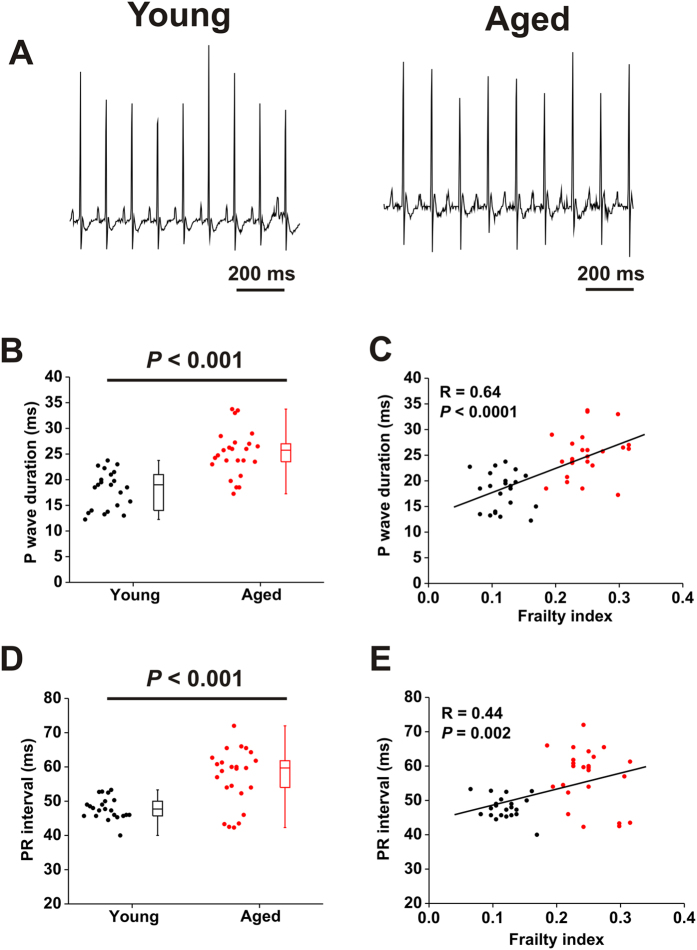
Effects of age and frailty on P wave duration and PR interval in anesthetized mice. (**A**) Representative surface ECG recordings in young and aged mice. (**B**) Summary of differences in P wave duration in young and aged mice. Data analyzed using Student’s *t*-test. (**C**) Linear regression analysis of P wave duration as a function of FI score for the same mice as used in panel B. (**D**) Summary of differences in PR interval in young and aged mice. Data analyzed using Student’s *t*-test. (**E**) Linear regression analysis of PR interval as a function of FI score for the same mice as used in panel D. For panels B and D *n* = 22 young mice and 24 aged mice. For panels C and E *n* = 46 mice.

Based on the evidence of increased predisposition to sustained AF and increased incidence of AV node block in aged mice we next measured P wave duration (a measure of atrial conduction time) and PR interval (a measure of conduction through the AV node) from ECG recordings in anesthetized young and aged mice (Fig. 4, Supplemental Table 4). P wave duration was increased (*P* < 0.001) in aged mice (Fig. 4B). Notably, there was substantial variability in both age groups so that mice of similar chronological age could have very different P wave durations, indicating that factors other than chronological age may impact atrial conduction time. To determine how P wave duration was impacted by frailty, P wave duration in each individual from both age groups was plotted as a function of each animal’s FI score (Fig. 4C). These data demonstrate that P wave duration is strongly correlated (*P* < 0.0001) with frailty.

PR interval was also increased (*P* < 0.001) in aged mice (Fig. 4D). Once again, there was substantial variability within age groups, particularly in the aged mice. Frailty analysis (Fig. 4E) illustrates that, similar to P wave duration, PR interval is correlated with FI score (*P* = 0.002). Thus, it becomes evident from these plots that young and aged mice with similar FI scores have similar measures of P wave duration and PR interval despite their differences in chronological age. Together, these analyses demonstrate that P wave duration and PR interval were graded with FI score such that these measures of atrial and AV node conduction fall along a continuum when measured as a function of frailty (i.e. health status).

We also assessed P wave duration and PR interval in young and aged mice after the application of atropine and propranolol to block muscarinic and β-adrenergic receptors respectively (Supplemental Fig. S1). This enables an assessment of atrial and AV node conduction independently of the autonomic nervous system^23^,^24^,^29^. P wave duration was increased (*P* < 0.05) following autonomic blockade in both young and aged mice. Furthermore, following blockade, P wave duration remained prolonged (*P* < 0.05) in aged mice vs. young mice (Supplemental Fig. S1B). Consistent with the data in Fig. 4, linear regression analysis demonstrates that P wave duration is correlated with FI score at baseline (*P* = 0.0001; Supplemental Fig. S1C) and after autonomic blockade (*P* = 0.02; Supplemental Fig. S1D). Similarly, following autonomic blockade, PR interval remained prolonged (*P* < 0.05) in aged mice vs. young mice (Supplemental Fig. S1E). Once again, scatter plots of PR interval as a function of FI score demonstrate PR interval is graded as a function of frailty at baseline (*P* = 0.001; Supplemental Fig. S1F) and after autonomic blockade (*P* = 0.002; Supplemental Fig. S1G). These data demonstrate that, independent of the autonomic nervous system, conduction across the atria and through the AV node becomes progressively slower as frailty increases.

Changes in P wave duration and PR interval are thought to be indicators of susceptibility to AF^30^,^31^. Furthermore, our data illustrate that AF severity is greater in older, more frail mice and that P wave duration and PR interval are graded by FI score. Accordingly, we examined whether AF duration demonstrated any relationships with P wave duration or PR interval in the mice that were induced into AF in our study (Supplemental Fig. S2). These measurements illustrate that AF duration was highly correlated with both P wave duration (*P* = 0.04) and PR interval (*P* = 0.003).

### Impacts of age and frailty on atrial conduction patterns and action potential morphology

**Figure 5:**
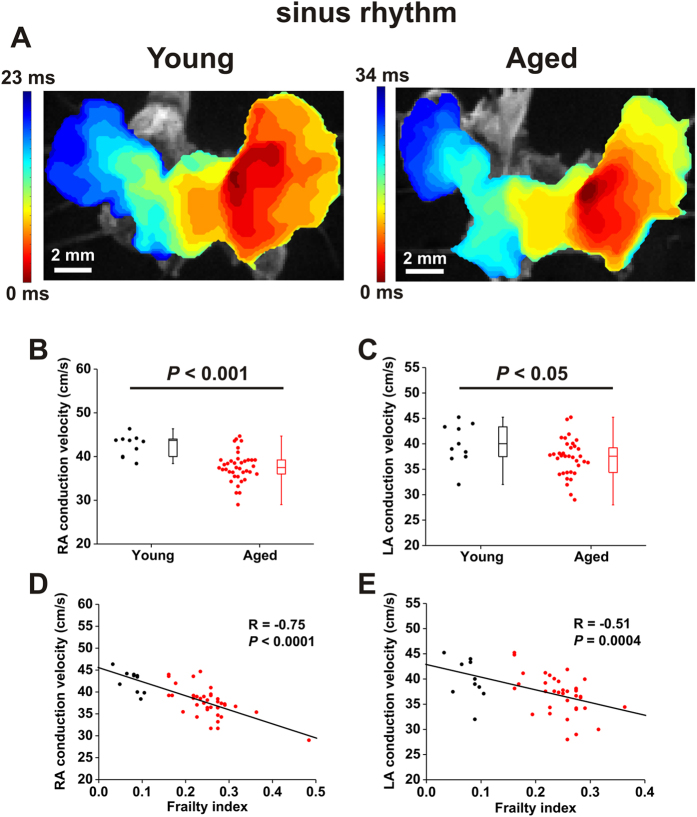
Effects of age and frailty on patterns of atrial conduction. (**A**) Representative activation maps in atrial preparations from a young mouse and an aged mouse. The right atrium is on the right side of the image. Red colour indicates the earliest activation time in the right atrial posterior wall. Time interval between isochrones is 1.25 ms. (**B** and **C**), Summary of the effects of age on right atrial (**B**) and left atrial (**C**) conduction velocity. Data analyzed using Student’s *t*-test; *n* = 10 young and 40 aged mice. (**D** and **E**), Linear regression analysis of right atrial (**D**) and left atrial (**E**) conduction velocity as a function of FI score; *n* = 50 mice for panels D and E.

To study how age and frailty affect patterns of electrical conduction in the atria we used high resolution optical mapping in isolated atrial preparations (Fig. 5) as we have done previously^23^,^25^,^26^. Representative activation maps (Fig. 5A) demonstrate that conduction initiates in the right atrial posterior wall (which corresponds to the location of the sinoatrial node^23^,^24^) and then spreads throughout the right and left atria. Note that in these examples conduction time across the atrial preparation is longer in the aged heart (34 ms) compared to the young heart (23 ms).

**Figure 6:**
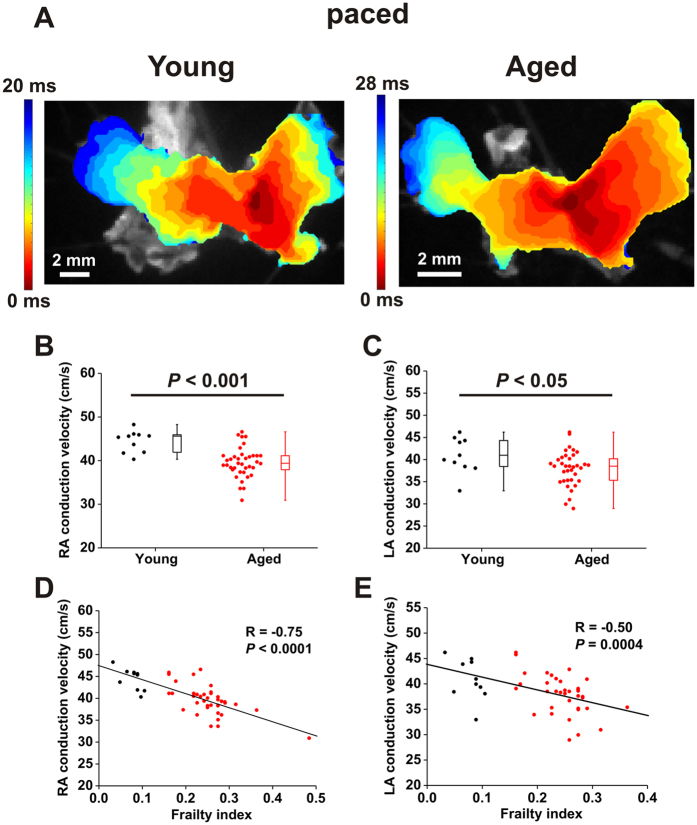
Effects of age and frailty on atrial conduction velocity in paced atrial preparations. (**A**) Representative activation maps in atrial preparations from a young mouse and an aged mouse paced at a fixed cycle length of 125 ms. (**B** and **C**) Summary of the effects of age on right atrial (**B**) and left atrial (**C**) conduction velocity. Data analyzed using Student’s *t*-test; *n* = 10 young and 40 aged mice. (**D** and **E**), linear regression analysis of right atrial (**D**) and left atrial (**E**) conduction velocity as a function of FI score; *n* = 50 mice.

We measured local conduction velocity (CV) in the right and left atria from these optical mapping studies. These measurements, performed in atrial preparations in sinus rhythm, demonstrate that CV was reduced in the right (*P* < 0.001; Fig. 5B) and left (*P* < 0.05; Fig. 5C) atria of aged mice. Similar to our *in vivo* findings, local CV in the atria showed substantial variability within age groups, especially in the aged mice. Accordingly, we next plotted local right and left atrial CV as a function of frailty. These data show that CV was highly correlated with FI score in the right (*P* < 0.0001; Fig. 5D) and left (*P* = 0.0004; Fig. 5E) atria. We also performed optical mapping studies in atrial preparations that were paced at a fixed cycle length (125 ms) in order to account for the possibility of rate dependent effects on atrial conduction (Fig. 6). The results from these experiments were very similar to the findings obtained from atrial preparations in sinus rhythm and further support the conclusion that right and left atrial conduction decreases with increasing FI score, including within an age group.

**Figure 7:**
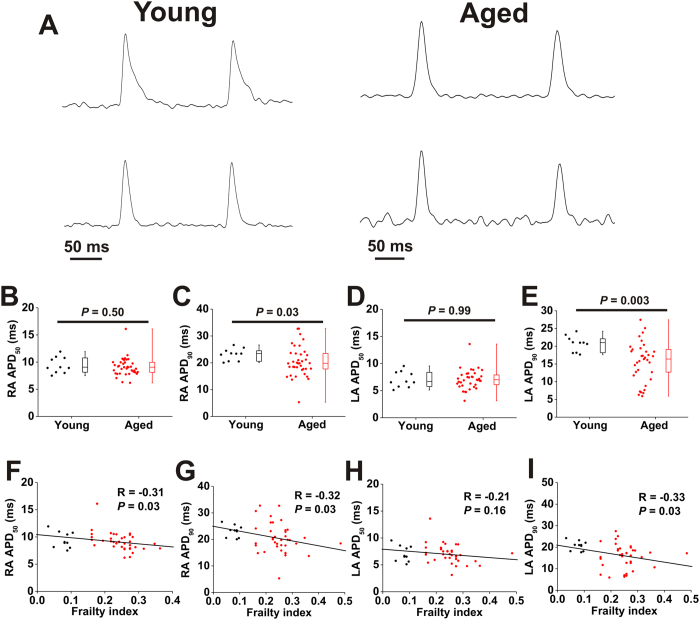
Effects of age and frailty on right and left atrial action potential morphology. (**A**) Representative optical APs from the right atrium (top) and left atrium (bottom) in young (left) and aged (right) mice. Right atrial APs were measured in the right atrial appendage while left atrial APs were measured in the left atrial appendage. (**B–E**), Summary data illustrating the effects of age on right atrial APD_50_ (**B**), right atrial APD_90_ (**C**), left atrial APD_50_ (**D**) and left atrial APD_90_ (**E**); data analyzed by Mann Whitney rank sum test; *n* = 10 young and 40 aged mice. (**F–I**), Linear regression analysis illustrating correlations between right atrial APD_50_ (**F**), right atrial APD_90_ (**G**), left atrial APD_50_ (**H**) or left atrial APD_90_ (**I**) and FI score; *n* = 50 mice.

Next, we measured the effects of age and frailty on optical action potential (AP) morphology in the right and left atria (Fig. 7). AP duration at 50% repolarization (APD_50_) was not different (*P* = 0.50; Fig. 7B) while APD_90_ was reduced (*P* = 0.03; Fig. 7C) in the right atrium of aged mice. We did not detect a difference in left atrial APD_50_ between age groups (*P* = 0.99; Fig. 7D), but left atrial APD_90_ was also reduced in aged mice (*P* = 0.003; Fig. 7E). Aged mice, in particular, demonstrated substantial variability in APD in the right and left atria. When analyzed by FI score, we found that right atrial APD_50_ (Fig. 7F), right atrial APD_90_ (Fig. 7G) and left atrial APD_90_ (Fig. 7I) were all correlated with (*P* = 0.03) and graded by frailty. These findings demonstrate that frailty analysis enabled us to identify differences in atrial CV and APD as a function of differences in health status separately from chronological age.

### Effects of age and frailty on atrial fibrosis

**Figure 8:**
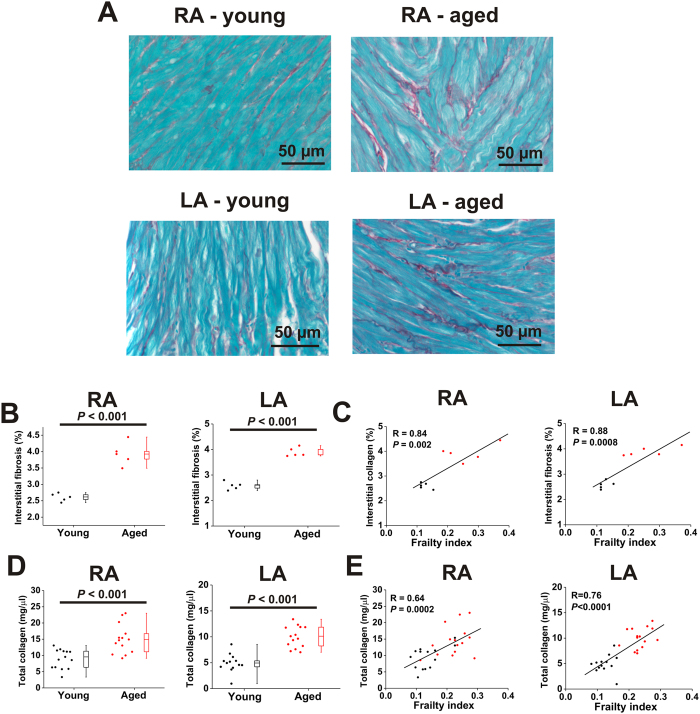
Effects of age and frailty on interstitial fibrosis and collagen content in the right and left atria. (**A**) Representative images demonstrating patterns of interstitial collagen deposition (red colour) in the right atrium (RA) and left atrium (LA) of young and aged mice. (**B**) Summary data quantifying right and left atrial fibrosis (measured from histological images as in panel A); data analyzed using Student’s *t*-test; *n* = 5 young and 5 aged hearts. (**C**) Linear regression analysis of interstitial fibrosis in the right and left atria as a function of FI score; *n* = 10 mice. (**D**) Effects of age on total collagen content in the right and left atria by hydroxyproline assay. Data analyzed using Student’s *t*-test; *n* = 14 young and 15 aged mice. (**E**) Linear regression analysis of total collagen content in the right and left atria as function of FI score, *n* = 29 mice.

Slow conduction and increased susceptibility to AF can be caused in part by structural remodelling in the atria due to the accumulation of collagen^4^,^32^. Accordingly, we assessed patterns of interstitial fibrosis in the right and left atria using picrosirius red staining^23^,^24^. Representative histological images (Fig. 8A) as well as summary data (Fig. 8B) demonstrate that interstitial fibrosis was increased in the right (*P* < 0.001) and left (*P* < 0.001) atria in aged mice. Furthermore, interstitial fibrosis was highly correlated with FI score in the right (*P* = 0.002) and left (*P* = 0.0008) atria such that atrial fibrosis was graded with frailty (Fig. 8C). Collagen content in the atria was also quantified using hydroxyproline assays. These measurements demonstrated that total collagen content was increased in the right (*P* < 0.001) and left (*P* < 0.001) atria of aged mice (Fig. 8D). Furthermore, frailty analysis illustrates that total collagen content in the right (*P* = 0.0002) and left (*P* < 0.001) atria was highly correlated with FI score (Fig. 8E). From these analyses it is clear that young and aged mice with similar FI scores have similar levels of atrial collagen content regardless of their substantial differences in chronological age.

We next investigated the mRNA expression patterns of genes involved in the regulation of fibrosis in the atria of aged mice and determined how they correlate with frailty. To determine if active collagen production was enhanced we measured the mRNA expression of collagen type I and collagen type III, which are major interstitial collagens in the myocardium^33^, in the right and left atria (Supplemental Fig. S3). These data demonstrate that there were no differences in expression of collagen I or collagen III in the right or left atria between young and aged mice. Furthermore, there were no significant correlations between collagen I or collagen III and FI score in the right or left atria. We also measured the expression of TGFβ and CTGF, which are both importantly involved in the production of collagens in the heart^34^,^35^, in the atria of young and aged mice (Supplemental Fig. S3). These data show that there were no differences in atrial TFGβ expression whether analyzed by age or by FI score. There were also no differences in atrial CTGF expression in young vs. aged mice or when measured as a function of frailty. Thus, our data show that atrial fibrosis is enhanced in the atria of aged and frail mice, but suggest that this is not due to actively increased collagen production via increased gene transcription.

We next evaluated whether fibrosis in the atria was associated with changes in regulation of the extracellular matrix by matrix metalloproteinases (MMPs) and tissue inhibitors of metalloproteinases (TIMPs). MMPs are involved in the processing and degradation of collagen (and other extracellular matrix proteins) while TIMPs function to inhibit the activity of MMPs^33^,^36^,^37^,^38^,^39^.

We initially focused on MMP2 and MMP9 as these are key gelatinases in the heart that play an important role in the degradation of collagens^38^. When measured as a function of age, MMP2 expression was not changed in the right atrium (*P* = 0.38), but was reduced (*P* < 0.001) in the left atrium of aged mice (Supplemental Fig. S4A). MMP2 expression was also negatively correlated (*P* = 0.0001) with FI score in the left atrium (Supplemental Fig. S4B). MMP9 expression was increased (*P* = 0.005) in the right atrium of aged mice, but not changed (*P* = 0.103) in the left atrium (Supplemental Fig. S4C). Interestingly, when analyzed as a function of frailty, MMP9 was positively correlated (*P* = 0.03) with FI score in the right atrium, but negatively correlated (*P* = 0.05) with FI score in the left atrium (Supplemental Fig. S4D).

We also measured the expression of TIMP1–4 (Supplemental Fig. S5) in the right and left atria as a function of age and FI score. When measured according to age we found that TIMP1 was increased (*P* = 0.001) in the left atrium of aged mice, TIMP2 was decreased (*P* = 0.04) in the left atrium of aged mice, TIMP3 was decreased in the right (*P* = 0.05) and left (*P* = 0.01) atria of aged mice and TIMP4 was increased (*P* = 0.002) in the right atrium of aged mice. When measured as a function of frailty, TIMP1 was positively correlated (*P* = 0.0003) with FI score in the left atrium, TIMP3 was negatively correlated with FI score in the right (*P* = 0.03) and left (*P* = 0.001) atria and TIMP4 was positively correlated (*P* = 0.01) with FI score in the right atrium.

**Figure 9:**
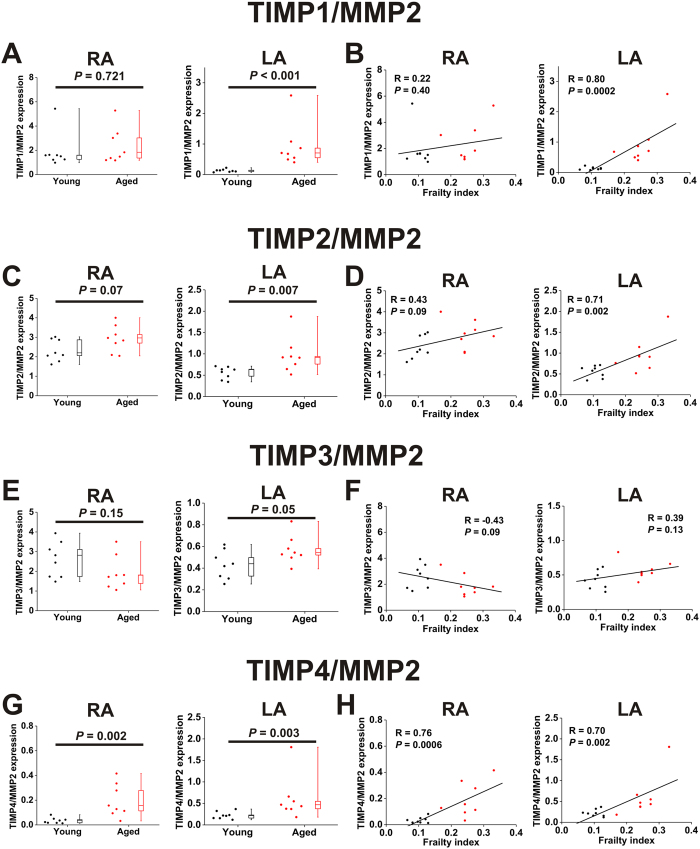
Ratios of expression of TIMPs to MMP2 in the right and left atria of young and aged mice. (**A**) Effects of age on the TIMP1/MMP2 mRNA expression ratio in the right (RA) and left (LA) atria. Data analyzed by Student’s *t*-test for the RA and Mann Whitney rank sum test for the LA; *n* = 8 young and 8 aged hearts. (**B**) Linear regression analysis illustrating correlations between the TIMP1/MMP2 expression ratio and FI score in the right and left atria; *n* = 16 hearts. (**C**) Effects of age on the TIMP2/MMP2 expression ratio in the right (RA) and left (LA) atria. Data analyzed by Student’s *t*-test for the RA and Mann Whitney rank sum test for the LA; *n* = 8 young and 8 aged hearts. (**D**) Linear regression analysis illustrating correlations between the TIMP2/MMP2 expression and FI score in the right and left atria; *n* = 16 hearts. (**E**) Effects of age on the TIMP3/MMP2 expression ratio in the right (RA) and left (LA) atria. Data analyzed by Student’s *t*-test; *n* = 8 young and 8 aged hearts. (**F**) Linear regression analysis illustrating correlations between the TIMP3/MMP2 expression ratio and FI score in the right and left atria; *n* = 16 hearts. (**G**) Effects of age on the TIMP4/MMP2 expression ratio in the right (RA) and left (LA) atria. Data analyzed by Mann Whitney rank sum test; *n* = 8 young and 8 aged hearts. (**H**) Linear regression analysis illustrating correlations between the TIMP4/MMP2 expression ratio and FI score in the right and left atria; *n* = 16 hearts.

Remodelling of the extracellular matrix is importantly determined by the balance between MMP and TIMP activities; therefore, we quantified the ratios between expression of TIMPs and MMPs as a function of age and frailty. The mRNA expression rations of TIMPs1-4 to MMP2 are presented in Fig. 9. Key differences include: TIMP1/MMP2 expression was increased (*P* < 0.001) in the left atrium of aged mice and positively correlated (*P* = 0.0002) with FI score in the left atrium, TIMP2/MMP2 expression was increased (*P* = 0.007) in the left atrium of aged mice and positively correlated (*P* = 0.002) with FI score in the left atrium, TIMP3/MMP2 was increased (*P* = 0.05) in the left atrium of aged mice, and TIMP4/MMP2 was increased in the right (*P* = 0.002) and left (*P* = 0.003) atria of aged mice as well as positively correlated with FI score in the right (*P* = 0.0006) and left atria (*P* = 0.002). There was also a trend towards an increase in TIMP2/MMP2 in the right atrium both as a function of age (*P* = 0.07) and FI score (*P* = 0.09).

**Figure 10:**
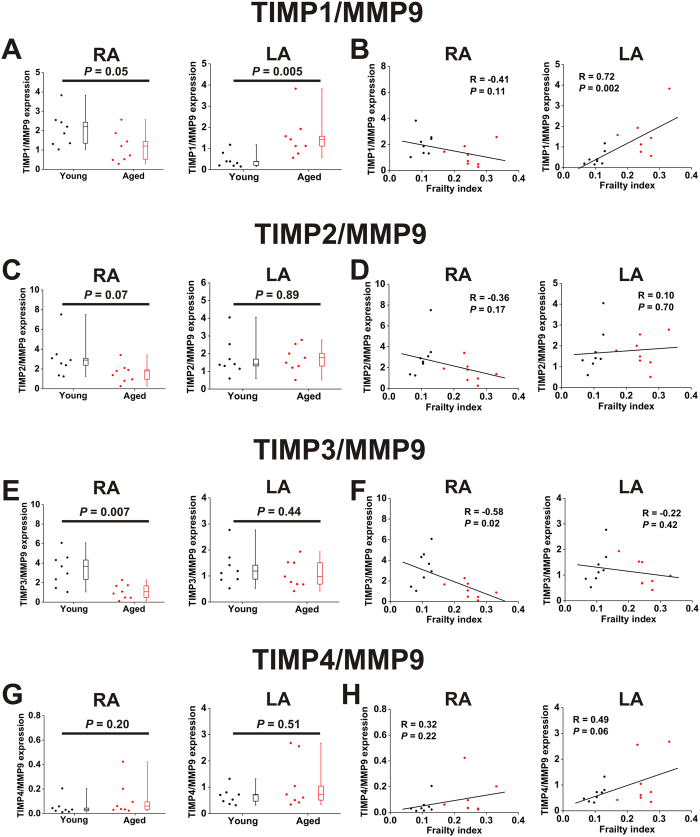
Ratios of expression of TIMPs to MMP9 in the right and left atria of young and aged mice. (**A**) Effects of age on the TIMP1/MMP9 mRNA expression ratio in the right (RA) and left (LA) atria. Data analyzed by Student’s *t*-test for the RA and Mann Whitney rank sum test for the LA; *n* = 8 young and 8 aged hearts. (**B**) Linear regression analysis illustrating correlations between the TIMP1/MMP9 expression ratio and FI score in the right and left atria; *n* = 16 hearts. (**C**) Effects of age on the TIMP2/MMP9 expression ratio in the right (RA) and left (LA) atria. Data analyzed by Mann Whitney rank sum test for the RA and Student’s *t*-test for the LA; *n* = 8 young and 8 aged hearts. (**D**) Linear regression analysis illustrating correlations between the TIMP2/MMP9 expression and FI score in the right and left atria; *n* = 16 hearts. (**E**) Effects of age on the TIMP3/MMP9 expression ratio in the right (RA) and left (LA) atria. Data analyzed by Mann Whitney rank sum test for the RA and Student’s *t*-test for the LA; *n* = 8 young and 8 aged hearts. (**F**) Linear regression analysis illustrating correlations between the TIMP3/MMP9 expression ratio and FI score in the right and left atria; *n* = 16 hearts. (**G**) Effects of age on the TIMP4/MMP9 expression ratio in the right (RA) and left (LA) atria. Data analyzed by Mann Whitney rank sum test; *n* = 8 young and 8 aged hearts. (**H**) Linear regression analysis illustrating correlations between the TIMP4/MMP9 expression ratio and FI score in the right and left atria; *n* = 16 hearts.

The mRNA expression ratios of TIMPs1-4 to MMP9 are presented in Fig. 10. Key differences include: TIMP1/MMP9 expression was increased (*P* = 0.005) in the left atrium of aged mice and positively correlated (*P* = 0.002) with FI score in the left atrium while TIMP3/MMP9 expression was reduced (*P* = 0.007) in the right atrium of aged mice and negatively correlated (*P* = 0.02) with FI score in the right atrium. There was a trend towards a positive correlation (*P* = 0.06) between TIMP4/MMP9 expression and FI score in the left atrium. Collectively, these data demonstrate a number of examples of increases in TIMP expression relative to MMP expression, which could explain, at least in part, the increase in interstitial fibrosis in the atria of aged and frail hearts (see Discussion).

## Discussion

We have investigated the impacts of age and frailty on atrial arrhythmogenesis as well as atrial structure and function. Our novel experiments demonstrate that aging is associated with electrophysiological alterations (i.e. changes in AP morphology) in the atria as well as enhanced atrial fibrosis. We further demonstrate that these changes are strongly correlated with and graded by FI score. These findings show that aging results in the creation of a substrate for atrial arrhythmias and that these changes are highly correlated with frailty. This indicates that the age-dependent development of a substrate for atrial arrhythmias is highly dependent on how successfully individuals age. Individuals that accumulated more health deficits, and that were therefore more frail, had more pronounced changes in atrial structure and function.

AF is well known to increase in incidence and severity (i.e. progression from paroxysmal to permanent AF) in the aged population^7^,^40^. Consistent with these clinical data we found that, although AF could be induced at similar rates in young vs. aged mice, the AF we observed in aged mice was substantially longer in duration compared to young mice. In fact, all AF observed in young mice was very brief, lasting much less than 5s before spontaneous reversion back to sinus rhythm. In contrast, when AF was induced in aged mice it was sustained (i.e. longer than 30s and up to minutes) in the majority of cases. Furthermore, we observed substantial rates of AV node block in aged mice, while young mice did not demonstrate any AV node block. These data clearly demonstrate that aged mice show a susceptibility to more sustained, longer lasting AF compared to young mice. Using our novel FI tool we also observed a clear difference in frailty (i.e. health status) in mice that were induced into AF, whereby the aged mice that demonstrated sustained AF had higher FI scores than the young mice that only exhibited brief episodes of AF. This indicates that sustained AF was associated with an increase in frailty.

Consistent with the increased duration of AF and occurrence of AV node block, we found that P wave duration (a measure of atrial conduction time) and PR interval (a measure of conduction through the AV node) were prolonged in aged mice in baseline conditions as well as after autonomic nervous system blockade. Interestingly, both measures showed substantial variability within age groups, particularly in the aged mice. This heteroscedasticity (i.e. increasing variability in the aged mice) in measures of atrial and AV node conduction is in agreement with the concept that not all individuals age at the same rate. In support of this, we assessed frailty in our young and aged mice and found that animals of the same chronological age can have very different FI scores. Strikingly, P wave duration and PR interval were highly correlated with FI score, regardless of chronological age. In fact, scrutiny of our data reveals that young and aged mice could have very similar P wave and PR interval durations when they had similar FI scores. Thus, these measures of atrial and AV node conduction fall along a continuum according to FI score, regardless of their similarities or differences in chronological age. This is consistent with the observation that FI scores in young and aged mice could overlap in some cases. Collectively, these analyses demonstrate that we were able to detect differences in health status (based on FI score) in young and aged mice and frailty is a strong predictor of these measures of atrial and AV node conduction. The results of these experiments are important because P wave duration and PR interval are strong predictors of susceptibility and severity of AF, including in aging populations^30^,^31^,^41^,^42^. Consistent with this idea, we found that when AF was induced in our study, the duration of AF was highly correlated with P wave duration and PR interval. This suggests that quantifying frailty could be a powerful approach to predicting alterations in atrial electrophysiology and the severity of AF. It should be noted that our measurements of atrial electrophysiology were made in anesthetized mice. Our data demonstrate that atrial electrophysiology (P wave duration, PR interval) was correlated with FI score at baseline and in the presence of autonomic blockade indicating that these changes are intrinsic to the atria. Nevertheless, future studies in conscious, freely moving mice, where heart rate would be expected to be higher in the absence of anesthetics, will be important.

A major advantage of our frailty approach in mice is that it enabled us to investigate the cellular and molecular mechanisms for frailty-related changes in atrial function. We used high resolution optical mapping to study the effects of age and frailty on atrial conduction patterns. These experiments clearly demonstrate that conduction is slowed throughout the atria in aged and frail hearts. Specifically, we found that local CV in the right and left atria was reduced in aged mice; however, as was the case in our *in vivo* studies, we observed substantial variability in atrial CV, especially in the aged mice, further suggesting that factors other than chronological age affect atrial CV. Indeed, we found that CV in the right and left atria was strongly negatively correlated with FI score whereby atrial CV was graded with frailty regardless of chronological age. We also measured optical APs in the right and left atria. As expected, APs in the right atrium were longer, on average, than the left atrium^43^. When measured as a function of age, we were able to detect reductions in APD_90_, but not APD_50_ in aged mice. Once again, there was a high degree of variability in these measurements, particularly in the aged mice. Interestingly, right atrial APD_50_, right atrial APD_90_ and left atrial APD_90_ were all negatively correlated with FI score. Thus, frailty was a strong predictor of changes in APD and, in some cases, was better able to identify changes than chronological age.

Reductions in atrial CV and APD can both increase the susceptibility to AF by shortening the wavelength of re-entry and creating a substrate for arrhythmia^4^,^44^. Our findings that atrial CVs and APDs are correlated with frailty is consistent with the observation that the AF we observed in aged, frail mice was longer (i.e. sustained) than younger, less frail mice. The effects of age on APD have been measured in prior studies with mixed results^45^. For example, prior investigations in aged dogs have reported that in the right atrium the AP is shorter in early repolarization but that APD_90_ is prolonged^46^,^47^. Studies in aged rabbits reported no change in APD_10_, but a prolongation of APD_90_^48^. In aged rats it has been reported that the right atrial AP is prolonged while the left atrial AP is shortened^49^ indicating that there may be regional differences in atrial electrophysiology during aging. Our studies in mice support the conclusion that APs are shortened in the right and left atria during aging, which is in agreement with studies showing that AP duration is shortened in persistent AF in humans^50^,^51^. Consistent with this idea prior studies have also reported that L-type Ca^2+^ currents are decreased while transient outward K^+^ currents are increased in aged atrial myocytes^45^,^52^. We have not investigated the specific ionic currents responsible for the changes APD we have observed in the present study. It will be important to consider this, as well as whether any changes in ion channel function correlate with FI score, in future studies. It will also be important to investigate whether aging and frailty are associated with changes in expression or function of connexins in the atria since gap junctions are important determinants of CV^53^ and mutations that impair gap junction function have previously been linked to AF^54^,^55^. Collectively, our data demonstrate that aging results in electrical remodelling in the atria that creates a substrate for AF to occur and that frailty is powerful predictor of these changes regardless of chronological age.

We also investigated whether differences in atrial arrhythmogenesis and electrophysiology were associated with changes in fibrosis. We observed an increase in right and left atrial fibrosis (based on histological assessments as well as measures of total collagen content) in aged mice, an observation that is consistent with prior studies^47^,^56^ and clinical data from human patients^57^. Importantly, we found that enhanced fibrosis in the right and left atria was highly correlated with FI score indicating that health status is a strong predictor of structural remodeling in the atria during the aging process. Structural remodeling is an important contributor to the slowing of conduction in the heart because enhanced collagen deposition can interfere with electrical conduction^58^,^59^. This is now thought to be a major contributor to the substrate for AF^4^,^57^. Our finding that structural remodeling is enhanced in frail hearts is consistent with our observations that atrial conduction velocities and duration of AF are also correlated with FI score.

The mechanisms leading to atrial structural remodeling during aging are not well understood. Initially, we investigated whether active collagen production, in association with increased gene expression, was altered as a function of age or frailty. We observed no differences in expression of collagens or profibrotic proteins such as TGFβ or CTGF^34^, whether analyzed by chronological age or by frailty. This suggests that enhanced atrial fibrosis in aged and frail hearts is not due to actively enhanced collagen production, which is consistent with prior investigations^60^. We also studied whether atrial fibrosis was associated with changes in atrial gene expression of MMPs and TIMPs, which play critical roles in maintenance and remodelling of the extracellular matrix^33^,^37^,^38^,^39^. Prior studies have shown that circulating levels of MMPs and TIMPs are altered in aging^61^, but few studies have assessed cardiac specific expression. Accordingly, we measured the mRNA expression of MMPs and TIMPs in the right and left atria and analyzed these data as a function of age and frailty. We observed differences in atrial expression of MMP2 and MMP9, which both play a role in the degradation of interstitial collagen^33^, as a function of age and frailty. Similarly, TIMPs 1, 3 and 4, but not TIMP2, showed expression changes in aged and/or frail hearts. These findings strongly support the hypothesis that atrial fibrosis in aged and frail hearts is at least partially due to changes in extracellular matrix remodelling by MMPs and TIMPs.

Remodeling of the extracellular matrix is dependent on the balance between MMP and TIMP activity^33^; therefore, we quantified the ratios of TIMPs1-4 to MMP2 and MMP9 in the atria as a function of chronological age and frailty. This approach revealed several important differences. Specifically, TIMP1/MMP2 and TIMP2/MMP2 were both increased in the left atrium (but not the right atrium) of aged and frail hearts. There was also a trend towards an increase in the TIMP2/MMP2 ratio in the right atrium of aged and frail hearts. TIMP4/MMP2 was clearly increased in the right and left atria as a function of age and frailty. We also observed increases in the TIMP1/MMP9 ratio in the left atrium of aged and frail hearts while TIMP3/MMP9 was reduced in the right atrium as a function of age and frailty. Collectively, these findings strongly support the emerging hypothesis that fibrosis in the aging heart is associated with changes in extracellular matrix remodelling by MMPs and TIMPs rather than increases in active collagen deposition^60^. Our observation that atrial fibrosis in aged and frail hearts is associated with changes in the balance between TIMP and MMP expression is consistent with prior studies of aging in the sinoatrial node^19^ and ventricular myocardium^22^,^60^,^62^,^63^. Increases in the expression ratio of TIMP to MMP, of which we observed several examples, would be expected to reduce MMP activity and could at least partially explain the increases in fibrosis and collagen content that we observed.

Our studies of regulators of the extracellular matrix focused on patterns of gene expression. It is important to note that gene expression is only one aspect of how these proteins may regulate the extracellular matrix, including during the aging process. These proteins, including TGFβ, CTGF, MMPs and TIMPs are regulated in complex ways^35^,^38^ and it is conceivable that the function of a number of these proteins may be altered as a function of age and/or frailty independently of changes (or lack thereof) in gene expression. Similarly, while we have identified changes in expression patterns for several MMPs and TIMPs that are consistent with the enhanced fibrosis we have observed in the atria of aged and frail mice, it will be important to follow up on these studies with measurements of collagenase and gelatinase activity to further investigate the roles of MMPs and TIMPs in aging related remodelling of the extracellular matrix and fibrosis. While changes in expression ratios between TIMPs and MMPs are indicative of a change in extracellular matrix degradation^19^,^22^ it must be recognized that all TIMPs can inhibit a large number of MMPs and that a number of MMPs are expressed in the heart^38^. Thus, in summary, our findings demonstrate that aged and frail hearts are characterized by enhanced atrial fibrosis and that MMPs and TIMPs are importantly involved in this process; however, this is a complex area of study and additional experiments will be required to better understand how MMPs and TIMPs regulate the extracellular matrix in the atria as a function of age and frailty as well as the links between atrial fibrosis and AF.

Our finding that frailty is powerful predictor of atrial structure, function and arrhythmogenesis has important clinical implications. For example, the patients that are enrolled in clinical trials are typically highly selected and are at a lower risk for adverse outcomes than patients from routine practice, potentially impacting the conclusions of trials^7^. In agreement with this, trials of oral anticoagulants for the prevention of stroke in elderly patients with AF have been criticised for not properly representing frail patients^64^. Our work demonstrates conclusively that frailty enables us to detect differences in health status regardless of chronological age and that structural and electrical changes in the atria that create a substrate for AF are strongly correlated with FI score.

Many common forms of cardiovascular disease (i.e. hypertension, hypertrophy, heart failure) occur preferentially in aged populations and many of these diseases also create a substrate for AF. Thus it is likely that both aging and concurrent cardiovascular disease can both contribute to the creation of a substrate for AF; however, as we have shown, the effects of aging on atrial structure and function are heterogeneous and highly related to an individual’s health status. This suggests that quantifying frailty, as can be done using an FI, can provide powerful insight into the degree of atrial remodelling in individual patients, which will have important implications for understanding a patient’s likelihood of developing AF. Accordingly, frailty should be an important consideration when treating elderly patients with AF and/or common forms of cardiovascular disease and when designing and interpreting therapeutic interventions for AF in aged populations.

In conclusion, the incidence and severity of AF increases with age and this represents a major clinical challenge. We have studied the effects of age on atrial structure, function and arrhythmogenesis and utilized a FI to determine how health status of individuals impacts atrial function. We demonstrate that aged mice exhibit electrical and structural remodelling in the atria, both of which could contribute to the creation a substrate for AF to occur. Furthermore, our novel approach to quantifying frailty enabled us to demonstrate that frailty is highly associated with changes in atrial function, including at the cellular and molecular levels. Specifically, we found that all changes in atrial electrophysiology and fibrosis were graded by FI score indicating that mice of the same chronological age have measureable differences in health status that have profound impacts on atrial physiology. Our studies further demonstrate that frailty manifests at the molecular level and that these changes correlate with alterations in atrial electrophysiology and arrhythmogenesis in the intact organism. Given that many common cardiovascular diseases, including AF, occur in aged populations, our findings have important implications for understanding the mechanisms of diseases in the elderly and suggest that frailty can be used to better understand the health status of similarly aged populations, which may have important implications for treatments strategies.

## Additional Information

**How to cite this article**: Jansen, H. J. *et al*. Atrial structure, function and arrhythmogenesis in aged and frail mice. *Sci. Rep.* **7**, 44336; doi: 10.1038/srep44336 (2017).

**Publisher's note:** Springer Nature remains neutral with regard to jurisdictional claims in published maps and institutional affiliations.

## Supplementary Material

Supplementary data
