# Supplementary material for: Atrial structure, function and arrhythmogenesis in aged and frail mice
Source: Sci Rep. 2017 Mar 14;7:44336. doi: 10.1038/srep44336 (PMC5349540; doi:10.1038/srep44336)
Supplement: Supplementary data [file srep44336-s1.pdf]

Atrial structure, function and arrhythmogenesis in aged and frail mice

Supplementary Information

Hailey J. Jansen[1,+] ,Motahareh Moghtadaei[1,+], Martin Mackasey[1], Sara A. Rafferty[1], Oleg Bogachev[1], John L. Sapp[1,3], Susan E. Howlett[2], Robert A. Rose[1,4,*]

[1]Department of Physiology and Biophysics
[2]Department of Pharmacology
[3]Division of Cardiology
[4]School of Biomedical Engineering
Faculty of Medicine
Dalhousie University
Halifax, Nova Scotia, Canada

[+] H. J. Jansen and M. Moghtadaei contributed equally to this work

*Corresponding author
Department of Physiology and Biophysics
Dalhousie University
Sir Charles Tupper Medical Building – Room 4J
5850 College Street
PO Box 15000
Halifax, Nova Scotia
Canada
B3H 4R2

Phone: 902-494-2268
Fax: 902-494-1685
Email: robert.rose@dal.ca

Supplemental Methods

In vivo electrophysiology and intracardiac programmed stimulation

Surface ECGs were measured in anesthetized mice (2% isoflurane inhalation) using 30 gauge subdermal needle electrodes (Grass Technologies). A 1.2 French octapolar electrophysiology catheter containing 8 electrodes spaced 0.5 mm apart (Transonic) was used for intracardiac pacing experiments. Correct catheter placement was ensured by obtaining a sole ventricular signal in the distal lead and a predominant atrial signal in the proximal lead. All stimulation pulses were given at 3 V for 2 ms, which enabled continuous capture and drive of cardiac conduction. Atrial (AERP) and atrioventricular node (AVERP) effective refractory period were measured using an 8 stimulus drive train (S1) at a cycle length of 100 ms followed by an extra stimulus (S2) at progressively shorter cycle lengths. Effective refractory periods were defined as the shortest S1-S2 interval allowing for capture in the region of interest. Inducibility of atrial fibrillation (AF) was studied using burst pacing in the right atrium as described previously[1]. Data were acquired using a Gould ACQ-7700 amplifier and Ponemah Physiology Platform software (Data Sciences International). Body temperature was monitored continuously via a rectal probe and maintained at 37°C with a heating pad.

High resolution optical mapping

To study patterns of electrical conduction in the atria we used high resolution optical mapping in atrial preparations as we have done previously[1-4]. To isolate our atrial preparation mice were administered a 0.2 ml intraperitoneal injection of heparin (1000 IU/ml) to prevent blood clotting and were then anesthetized by isoflurane inhalation and cervically dislocated. Hearts were excised into Krebs solution (35°C) containing (in mM): 118 NaCl, 4.7 KCl, 1.2 KH_2PO_4, 12.2 $MgSO_4$, 1 $CaCl_2$, 25 $NaHCO_3$, 11 glucose and bubbled with 95% O_2/5% CO_2 in order to maintain a pH of 7.4. The atria were dissected

away from the ventricles and pinned in a dish with the endocardial surface facing upwards (towards the imaging equipment). With this preparation conduction can be observed from its point of initiation in the right atrial posterior wall (i.e. the sinoatrial node[1,3,4]) and we can map conduction throughout the right and left atria.

The atrial preparation was superfused continuously with Krebs solution (37°C) bubbled with 95% O_2/5% CO_2 and allowed to equilibrate for at least 30 min. During this time the preparation was treated with the voltage sensitive dye di-4-ANEPPS (10 µM) and blebbistatin (10 µM) was added to the superfusate to suppress contractile activity[5,6]. Blebbistatin was present throughout the duration of the experiments in order to prevent motion artifacts during optical mapping. Experiments were performed in sinus rhythm so that the cycle length (i.e. beating rate) of the atrial preparation was free to change as well as in atrial preparations paced at a fixed cycle length of 125 ms in order to study electrical conduction independently of changes in cycle length. The pacing electrode was placed near the opening of the superior vena cava.

Di-4-ANEPPS loaded atrial preparations were illuminated with light at a wavelength of 520 – 570 nM using an EXFO X-cite fluorescent light source (Lumen Dynamics). Emitted fluorescent light (590 – 640 nm) was captured using a high speed EMCCD camera (Evolve 128, Photometrics). In these studies the spatial resolution of each pixel was 80 x 80 µM and data were acquired at 800 frames/s using Metamorph software (Molecular Devices). Magnification was constant in all experiments and no pixel binning was used.

All optical data were analyzed using custom software written in Matlab. Analyses included pseudocolor electrical activation maps, which were generated from measurements of activation time at individual pixels. In all cases background fluorescence was subtracted. Local conduction velocity (CV) was quantified specifically in the right and left atria using an established approach previously described[3,4,7,8]. Briefly, activation times at each pixel from a 7 x 7 pixel array were determined and fit to a plane using the least squares fit method. The direction

on this plane that is increasing the fastest represents the direction that is perpendicular to the wavefront of electrical propagation and the maximum slope represents the inverse of the speed of conduction in that direction. Thus, using this method, we computed maximum local CV vectors in the right and left atria. With pixel dimensions of 80 x 80 μM, the area of the 7 x 7 pixel array was 560 x 560 μM. Optical action potential data were obtained by measuring changes in fluorescence as a function of time at individual pixels within the right and left atria as we have described previously[3,4].

Quantitative PCR

Quantitative gene expression in the right and left atria was performed as we described previously[9,10]. Intron spanning primers were designed for collagen I (*col1a*), collagen III (*col3a*), matrix metalloproteinase 2 (MMP2), MMP9, tissue inhibitor of metalloproteinase 1 (TIMP1), TIMP2, TIMP3, TIMP4, transforming growth factor β (TGFβ) and connective tissue growth factor (CTGF). β-actin (*Actb*) and GAPDH were used as reference genes. All primer sequences are provided in Supplemental Table 2.

Following synthesis (Sigma-Aldrich, Invitrogen) primers were reconstituted in nuclease free water at a concentration of 100 nM and stored at -20°C. All primer sets were validated in order to determine optimal annealing temperature as well as confirmation of ideal amplification efficiency (between 90-110% copy efficiency per cycle).

RNA was extracted in PureZOL™ RNA isolation reagent according to kit instructions (Aurum Total RNA Fatty and Fibrous Tissue Kit, Bio-Rad). RNA was eluted in 40 μl of elution buffer from the spin column. RNA concentrations were determined using a Qubit fluorometer (Invitrogen) and first strand synthesis reactions were performed using the iScript cDNA synthesis kit (BioRad) according to kit instructions. The Experion™ Automated Electrophoresis System (Bio-Rad) was used to assess RNA quality by observing the 28S and 18S rRNA subunits prior to first strand synthesis. Lack of genomic DNA contamination was verified by

reverse transcription (RT)-PCR using a no RT control.

RT-qPCR using BRYT green dye (Promega) was used to assess gene expression. Following RNA extraction, cDNA was synthesized and 10 µl reactions were performed with 5.6 µl of SYBR green dye, 4 µl cDNA template (at the appropriate dilution), and 0.4 µl of primers. Primers were used at a concentration of 10 nM. Reactions were carried out using the CFX384 Touch™ Real-Time PCR Detection System (Bio-Rad). Amplification conditions were as follows: 95°C for 2 minutes to activate Taq polymerase, followed by 39 cycles of denaturation at 95°C for 15 seconds, annealing at 60°C for 30 seconds, and extension at 72°C for 30 seconds. Melt curve analysis was performed from 65-95°C in 0.5°C increments. Single amplicons with appropriate melting temperatures and sizes were detected. Data were analyzed using the $2^{-\Delta\Delta C_T}$ method by which the expression values are determined relative to an internal control and normalized to both GAPDH and β-actin.

Supplemental Results

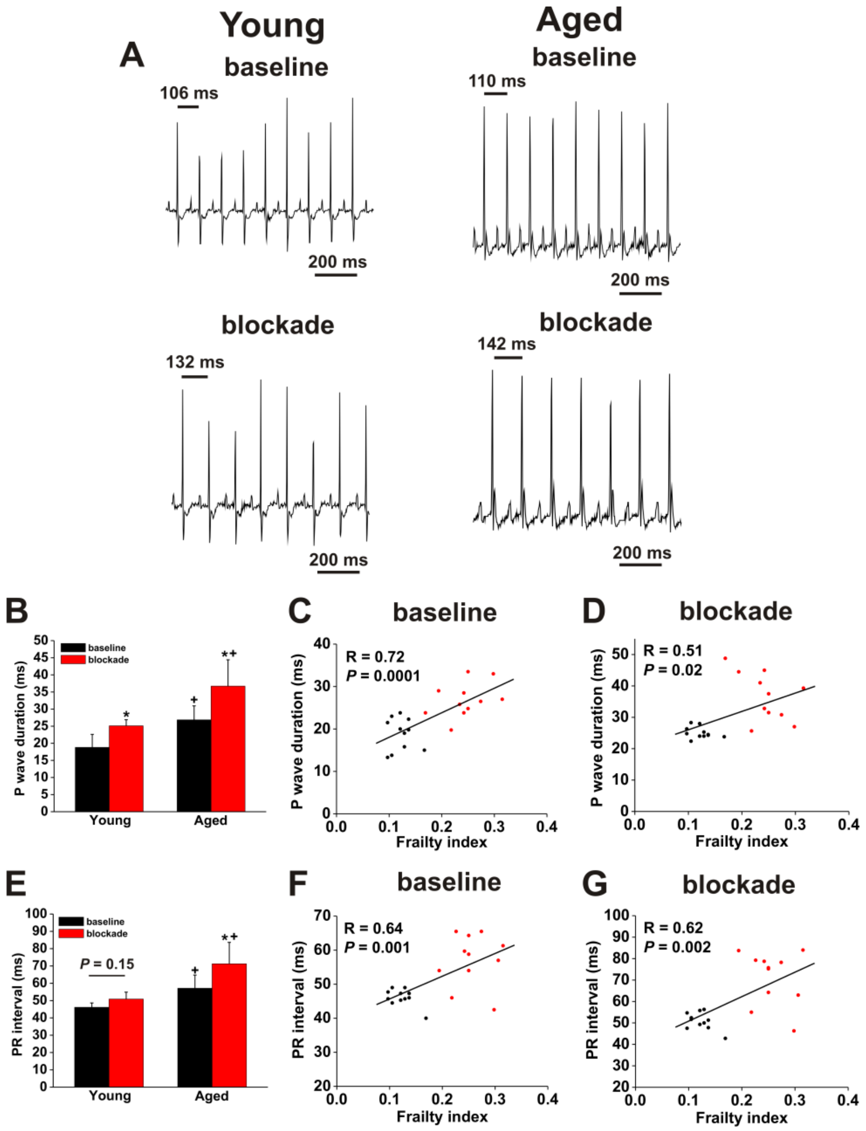

Figure S1. Effects of age and frailty on P wave duration and PR interval following autonomic nervous system blockade in anesthetized mice. A, Representative ECG recordings at baseline and following autonomic blockade by intraperitoneal injection of atropine (10 mg/kg) and propranolol (10 mg/kg). RR intervals are indicated in each recording. B, Summary of the effects of autonomic blockade on P wave duration in young and aged mice. **P*<0.05 vs baseline, $^{+}$*P*<0.05 vs. young by two way ANOVA with Tukey's posthoc test; *n*=11 young and 11 aged mice. C and D, Linear regression analysis of P wave duration as a function of FI score at baseline (C) and after autonomic blockade (D) for the same mice as used in panel B (*n*=22; correlation coefficients obtained using Pearson's correlation). E, Summary of the effects of autonomic blockade on PR interval in young and aged mice. **P*<0.05 vs baseline, $^{+}$*P*<0.05 vs. young by two way ANOVA with Tukey's posthoc test; *n*=11 young and 11 aged mice. F and G, Linear regression analysis of PR interval as a function of FI score at baseline (F) and after autonomic blockade (G) for the same mice as used in panel E (*n*=22; correlation coefficients obtained using Pearson's correlation).

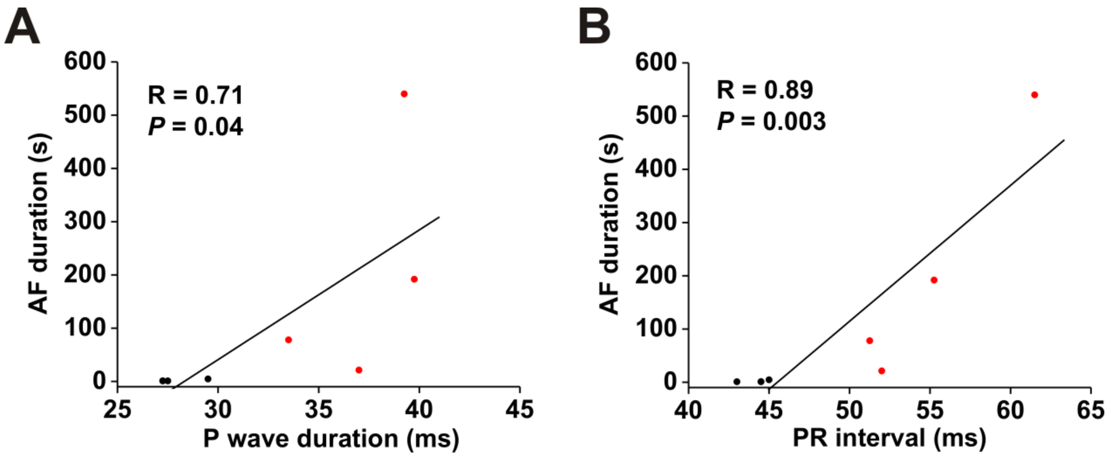

Figure S2. A, Correlation between P wave duration and duration of AF in young and aged mice that were induced into AF. B, Correlation between PR interval and AF duration in young and aged mice that were induced into AF. In both plots young mice (*n*=4) are shown in black symbols and aged mice (*n*=4) are shown in red symbols.

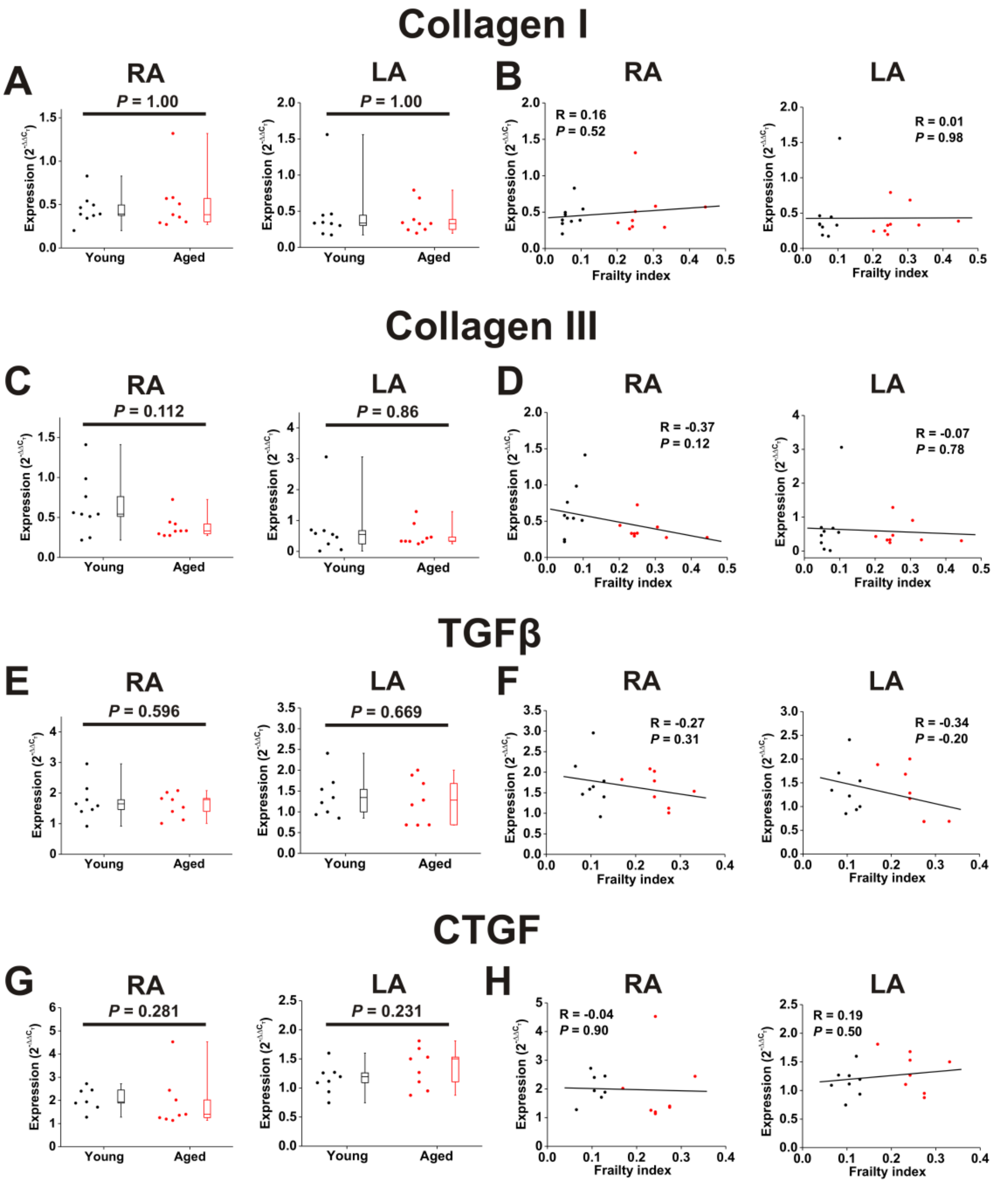

Figure S3. Expression of collagen I, collagen III, transforming growth factor β and connective tissue growth factor in the right and left atria of young and aged mice. A, Effects of age on collagen I mRNA expression in the right (RA) and left (LA) atria. Data analyzed by Mann Whitney rank sum test; *n*=8 young and 8 aged hearts. B, Linear regression analysis illustrating correlations between collagen I expression and FI score in the right and left atria; *n*=16 hearts. C, Effects of age on collagen III mRNA expression in the right (RA) and left (LA) atria. Data analyzed by Mann Whitney rank sum test; *n*=9 young and 9 aged hearts. D, Linear regression analysis illustrating correlations between collagen III expression and FI score in the right and left atria; *n*=18 hearts. E, Effects of age on TGFβ mRNA expression in the right (RA) and left (LA) atria. Data analyzed by Student's *t*-test; *n*=8 young and 8 aged hearts. F, Linear regression analysis illustrating correlations between TGFβ expression and FI score in the right and left atria; *n*=16 hearts. G, Effects of age on CTGF mRNA expression in the right (RA) and left (LA) atria. Data analyzed by Student's *t*-test; *n*=8 young and 8 aged hearts. H, Linear regression analysis illustrating correlations between CTGF expression and FI score in the right and left atria; *n*=16 hearts.

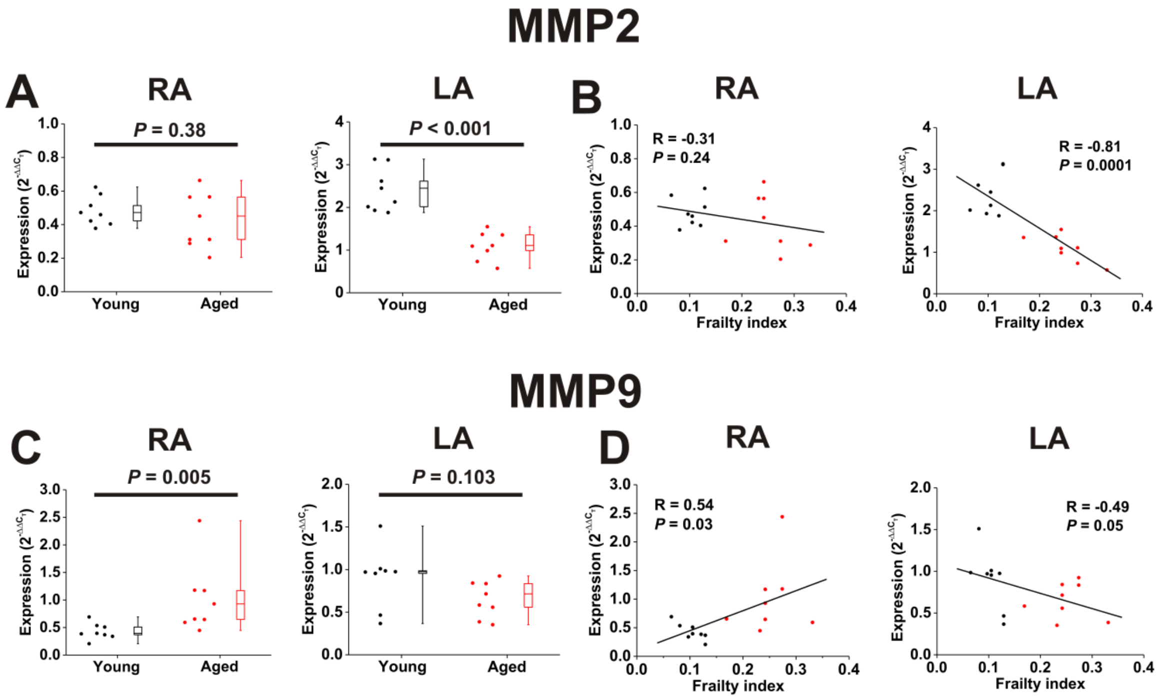

Figure S4. Expression of matrix metalloproteinases 2 and 9 in the right and left atria of young and aged mice. A, Effects of age on MMP2 mRNA expression in the right (RA) and left (LA) atria. Data analyzed by Mann Whitney rank sum test for the RA and Student's *t*-test for the LA; *n*=8 young and 8 aged hearts. B, Linear regression analysis illustrating correlations between MMP2 expression and FI score in the right and left atria; *n*=16 hearts. C, Effects of age on MMP9 mRNA expression in the right (RA) and left (LA) atria. Data analyzed by Mann Whitney rank sum test for the RA and Student's *t*-test for the LA; *n*=8 young and 8 aged hearts. D, Linear regression analysis illustrating correlations between MMP9 expression and FI score in the right and left atria; *n*=16 hearts.

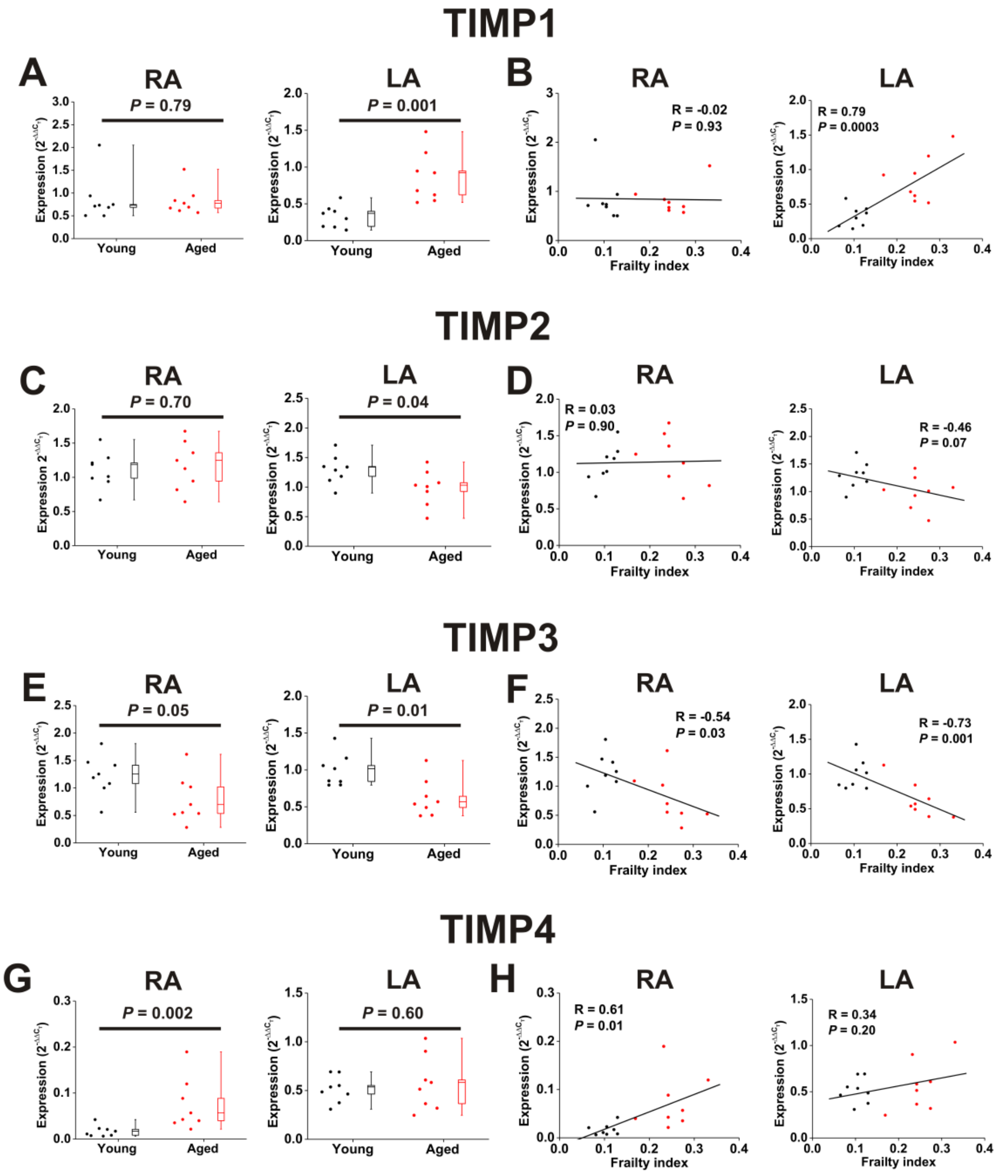

Figure S5. Expression of tissue inhibitors of metalloproteinases 1-4 in the right and left atria of young and aged mice. A, Effects of age on TIMP1 mRNA expression in the right (RA) and left (LA) atria. Data analyzed by Mann Whitney rank sum test for the RA and Student's *t*-test for the LA; *n*=8 young and 8 aged hearts. B, Linear regression analysis illustrating correlations between TIMP1 expression and FI score in the right and left atria; *n*=16 hearts. C, Effects of age on TIMP2 mRNA expression in the right (RA) and left (LA) atria. Data analyzed by Student's *t*-test; *n*=8 young and 8 aged hearts. D, Linear regression analysis illustrating correlations between TIMP2 expression and FI score in the right and left atria; *n*=16 hearts. E, Effects of age on TIMP3 mRNA expression in the right (RA) and left (LA) atria. Data analyzed by Student's *t*-test for the RA and Mann Whitney rank sum test for the LA; *n*=8 young and 8 aged hearts. F, Linear regression analysis illustrating correlations between TIMP3 expression and FI score in the right and left atria; *n*=16 hearts. G, Effects of age on TIMP4 mRNA expression in the right (RA) and left (LA) atria. Data analyzed by Mann Whitney rank sum test for the RA and Student's *t*-test for the LA; *n*=8 young and 8 aged hearts. H, Linear regression analysis illustrating correlations between TIMP4 expression and FI score in the right and left atria; *n*=16 hearts.

Supplemental Table 1: Mouse frailty assessment form

Date: ____________________

Mouse #: ________________ **Date of Birth:** _______________ **Sex:** F M
Body weight (g): ___________________ **Body surface temperature (°C):** ________________________

Rating: 0 = absent 0.5 = mild 1 = severe

				**NOTES:**
**Integument:**				
Alopecia	0	0.5	1	
Loss of fur colour	0	0.5	1	
Dermatitis	0	0.5	1	
Loss of whiskers	0	0.5	1	
Coat condition	0	0.5	1	
**Physical/Musculoskeletal:**				
Tumours	0	0.5	1	
Distended abdomen	0	0.5	1	
Kyphosis	0	0.5	1	
Tail stiffening	0	0.5	1	
Gait disorders	0	0.5	1	
Tremor	0	0.5	1	
Forelimb grip strength	0	0.5	1	
Body condition score	0	0.5	1	
**Vestibulocochlear/Auditory:**				
Vestibular disturbance	0	0.5	1	
Hearing loss	0	0.5	1	
**Ocular/Nasal:**				
Cataracts	0	0.5	1	
Corneal opacity	0	0.5	1	
Eye discharge/swelling	0	0.5	1	
Microphthalmia	0	0.5	1	
Vision loss	0	0.5	1	
Menace reflex	0	0.5	1	
Nasal discharge	0	0.5	1	
**Digestive/Urogenital:**				
Malocclusions	0	0.5	1	
Rectal prolapse	0	0.5	1	
Vaginal/uterine/penile prolapse	0	0.5	1	
Diarrhoea	0	0.5	1	
**Respiratory system:**				
Breathing rate/depth	0	0.5	1	
**Discomfort:**				
Mouse Grimace Scale	0	0.5	1	
Piloerection	0	0.5	1	

- **Temperature score:** __________
- **Body weight score:** _____________

Total Score/ Max Score:

© Susan E. Howlett, 2013

Deficits in body mass and body temperature were scored based on deviation from the mean of young or aged mice[11,12] (see methods).

Supplemental Table 2: Quantitative PCR primers

Gene of Interest	Forward Primer (5' → 3')	Reverse Primer (5' → 3')	Amplicon Length
TIMP1	CAGATACCATGATGGCCCCC	CGCTGGTATAAGGTGGTCTCG	190
TIMP2	CCAGAAGAAGAGCCTGAACCA	GTCCATCCAGAGGCACTCATC	112
TIMP3	GGCCTCAATTACCGCTACCA	CTGATAGCCAGGGTACCCAAAA	135
TIMP4	TGCAGAGGGAGAGCCTGAA	GGTACATGGCACTGCATAGCA	80
MMP2	CCACGTGACAAGCCCATGGGGCCC	GCAGCCTAGCCAGTCGGATTTGATG	486
MMP9	TCGCGTGGATAAGGAGTTCTC	ATGGCAGAAATAGGCTTTGTCTTG	82
TGFβ1	CGAGGTGACCTGGGCACCATCCATGAC	CTGCTCCACCTTGGGCTTGCGACCCAC	405
CTGF	TCCCGAGAAGGGTCAAGCT	TCCTTGGGCTCGTCACACA	222
Col1a	GCGGACTCTGTTGCTGCTTGC	GACCTGCGGGACCCCTTTGT	125
Col3a	AGATCCGGGTCCTCCTGGCATTC	CTGGTCCCGGATAGCCACCCAT	194
GAPDH	AATGGGGTGAGGCCGGTGCT	CACCCTTCAAGTGGGCCCCG	87
β-Actin	AGCCATGTACGTAGCCATCC	TCTCAGCTGTGGTGGTGAAG	227

Supplemental Table 3: Duration of arrhythmia in young and aged mice that were induced into atrial fibrillation

	Young	Aged
< 5s	100% (4/4)	0% (0/4)
5-30 s	0% (0/4)	25% (1/4)
>30 s	0% (0/4)	75% (3/4)

Numbers in parentheses indicate the number of mice in each group.

Supplemental Table 4: ECG intervals and intracardiac ECG parameters in young and aged mice

	Young	Aged	*P* value
Heart rate (beats/min)	553 ± 26	514 ± 53*	0.005
P wave (ms)	18.0 ± 3.7	25.2 ± 3.5*	<0.001
P-R interval (ms)	47.9 ± 3.2	57.0 ± 8.3*	<0.001
AERP (ms)	30.9 ± 5.0	34.1 ± 7.7	0.23
AVERP (ms)	46.6 ± 6.3	40.8 ± 12.3	0.11

Data are means ± SD; *n* = 22 young and 24 aged mice. *P<0.05 vs. young by Student's *t*-test. Abbreviations: AERP, atrial effective refractory period; AVERP, atrioventricular node effective refractory period.

References

1. Egom, E. E. *et al.* Impaired sinoatrial node function and increased susceptibility to atrial fibrillation in mice lacking natriuretic peptide receptor C. *J Physiol* **593**, 1127-46 (2015).
2. Krishnaswamy, P. S. *et al.* Altered parasympathetic nervous system regulation of the sinoatrial node in Akita diabetic mice. *J Mol Cell Cardiol* **82**, 125-35 (2015).
3. Azer, J., Hua, R., Krishnaswamy, P. S. & Rose, R. A. Effects of natriuretic peptides on electrical conduction in the sinoatrial node and atrial myocardium of the heart. *J Physiol* **592**, 1025-45 (2014).
4. Hua, R. *et al.* Effects of Wild-Type and Mutant Forms of Atrial Natriuretic Peptide on Atrial Electrophysiology and Arrhythmogenesis. *Circ Arrhythm Electrophysiol* **8**, 1240-54 (2015).
5. Fedorov, V. V. *et al.* Complex interactions between the sinoatrial node and atrium during reentrant arrhythmias in the canine heart. *Circulation* **122**, 782-9 (2010).
6. Farman, G. P. *et al.* Blebbistatin: use as inhibitor of muscle contraction. *Pflugers Arch* **455**, 995-1005 (2008).
7. Nygren, A., Lomax, A. E. & Giles, W. R. Heterogeneity of action potential durations in isolated mouse left and right atria recorded using voltage-sensitive dye mapping. *Am J Physiol Heart Circ Physiol* **287**, H2634-43 (2004).
8. Morley, G. E. *et al.* Characterization of conduction in the ventricles of normal and heterozygous Cx43 knockout mice using optical mapping. *J Cardiovasc Electrophysiol* **10**, 1361-75 (1999).
9. Springer, J. *et al.* The natriuretic peptides BNP and CNP increase heart rate and electrical conduction by stimulating ionic currents in the sinoatrial node and atrial myocardium following activation of guanylyl cyclase-linked natriuretic peptide receptors. *J Mol Cell Cardiol* **52**, 1122-34 (2012).
10. Hua, R., Adamczyk, A., Robbins, C., Ray, G. & Rose, R. A. Distinct patterns of constitutive phosphodiesterase activity in mouse sinoatrial node and atrial myocardium. *PLoS One* **7**, e47652 (2012).
11. Moghtadaei, M. *et al.* The impacts of age and frailty on heart rate and sinoatrial node function. *J Physiol* **594**, 7105-26 (2016).
12. Whitehead, J. C. *et al.* A Clinical Frailty Index in Aging Mice: Comparisons With Frailty Index Data in Humans. *J Gerontol A Biol Sci Med Sci* (2013).
